# Supplementation with soluble or insoluble rice-bran fibers increases short-chain fatty acid producing bacteria in the gut microbiota *in vitro*

**DOI:** 10.3389/fnut.2024.1304045

**Published:** 2024-05-10

**Authors:** Karley K. Mahalak, LinShu Liu, Jamshed Bobokalonov, Adrienne B. Narrowe, Jenni Firrman, Kyle Bittinger, Weiming Hu, Steven M. Jones, Ahmed M. Moustafa

**Affiliations:** ^1^Dairy and Functional Foods Research Unit, Eastern Regional Research Center, Agricultural Research Service, United States Department of Agriculture, Wyndmoor, PA, United States; ^2^V. I. Nikitin Institute of Chemistry, National Academy of Sciences, Dushanbe, Tajikistan; ^3^Division of Gastroenterology, Hepatology, and Nutrition, The Children’s Hospital of Philadelphia, Philadelphia, PA, United States; ^4^Department of Pediatrics, Perelman School of Medicine, University of Pennsylvania, Philadelphia, PA, United States

**Keywords:** gut microbiota, rice bran, *Bifidobacterium*, short-chain fatty acids, prebiotics

## Abstract

**Introduction:**

Studies have shown that a diet high in fiber and prebiotics has a positive impact on human health due largely to the fermentation of these compounds by the gut microbiota. One underutilized source of fiber may be rice bran, a waste product of rice processing that is used most frequently as an additive to livestock feed but may be a good source of fibers and other phenolic compounds as a human diet supplement. Previous studies focused on specific compounds extracted from rice bran showed that soluble fibers extracted from rice bran can improve glucose response and reduce weight gain in mouse models. However, less is known about changes in the human gut microbiota in response to regular rice bran consumption.

**Methods:**

In this study, we used a Simulator of the Human Intestinal Microbial Ecology (SHIME®) to cultivate the human gut microbiota of 3 different donors in conditions containing either soluble or insoluble fiber fractions from rice bran. Using 16S rRNA amplicon sequencing and targeted metabolomics via Gas Chromatography-Mass Spectrometry, we explored how gut microbial communities developed provided different supplemental fiber sources.

**Results:**

We found that insoluble and soluble fiber fractions increased short-chain fatty acid production, indicating that both fractions were fermented. However, there were differences in response between donors, for example the gut microbiota from donor 1 increased acetic acid production with both fiber types compared with control; whereas for donors 2 and 3, butanoic acid production increased with ISF and SF supplementation. Both soluble and insoluble rice bran fractions increased the abundance of *Bifidobacterium* and Lachnospiraceae taxa.

**Discussion:**

Overall, analysis of the effect of soluble and insoluble rice bran fractions on the human *in vitro* gut microbiota and the metabolites produced revealed individually variant responses to these prebiotics.

## 1 Introduction

Regular intake of dietary fiber is beneficial for human health due in part to reduced risks of cardiovascular disease, diabetes, colorectal cancers, and more (1–5). Dietary fibers that are found in plant-based foods and cereals including wheat, oat, millet, and rice are of particular interest due to their reported beneficial properties (6, 7). Dietary fibers remain unchanged through the digestive tract until they reach the colon, where they are then fermented by the gut microbiome (8). This fermentation process promotes a healthy mucosal barrier in the colon and an increase in the production of short-chain fatty acids (SCFAs), among other beneficial health effects (9). SCFAs absorbed by the human cells lining the intestine as an energy source, and contribute to overall health by functioning as carbon sources for the colonocytes (butyrate) and liver cells (acetic acid and propanoic acid) (10). Many studies have shown the beneficial effects of SCFAs on the body, including anti-inflammatory, anti-obesity, and immunoregulatory effects (10, 11). The ability of dietary fibers to aid against metabolic and other disorders is often attributed to their ability to regulate digestion, absorption of nutrients, and metabolism (2).

It is important to distinguish between dietary fiber types when looking at their effects on health. Two main fiber types, categorized as soluble and insoluble fibers, are important components of a healthy diet. Soluble fibers are quickly fermented by the gut microbiota in the colon, can increase satiety, and reduce the speed of movement through the large intestine (1, 12). Conversely, insoluble fibers increase the rate of passage through the large intestine and contribute to fecal bulking, however, there is less fermentation associated with insoluble fibers in the colon (13). While soluble fibers are well known for their fermentability by the gut microbiota, and consequently how they increase the production of SCFAs by the gut microbiota, less is known about insoluble fibers. In mouse studies, insoluble fibers have been shown to reduce weight gain and cholesterol, as well as increase SCFA production and modify the abundance of particular gut microbial community members (14). Insoluble fibers from bamboo have also been shown to increase SCFA production after 24 h of *in vitro* batch fermentation (15). Insoluble fibers extracted from soy husk and subjected to 48 h of *in vitro* batch fermentation by human fecal samples significantly changed the gut microbial community composition at the phylum level (16). Taken together, these findings indicate that insoluble fiber impacts the gut microbiota in a more meaningful way than was previously thought.

Rice is a staple food source around the world, with consumption increasing in the Americas due to the globalization of food (17). Unprocessed, whole-grain rice is considered healthier, as it contains more fibers and polyphenols, which are associated with the health benefits of rice and other whole-grain cereals. The milling of rice removes the outer layer of the rice grain, which is termed rice bran. Due to its high fat content, particularly free fatty acids, and the lipase enzyme, rice bran requires processing before use in human consumption to ensure it does not become rancid, an intensive process called stabilization (18). Due to the necessity of this process for human consumption, rice bran is often used instead as livestock feed as a bulking agent (19). However, if there are suitably notable beneficial health effects of rice bran extracts on gut health, there could be increased interest in its use as a food supplement.

Previous studies on the effect of rice bran components on the human gut microbiome have demonstrated that both of the soluble fiber components increased SCFA production *in vitro* in a short-term batch fermentation model. In those studies, rice bran modified the microbial community composition but did not increase the abundance of *Bifidobacterium* and *Lactobacillus* species, which did increase with other fiber treatments, such as fructooligosaccharides (20, 21). *In vivo* studies using dietary interventions with mouse models showed that phenolics found in the fibers from rice bran had antihyperglycemic effects, and in an irritable bowel disease (IBD) model, fermented rice bran helped alleviate symptoms of IBD (22–24).

The beneficial effects of dietary fiber have been studied in relation to overall health as well as gut health for many years. However, there are few studies directly comparing fiber types from the same plant on the gut microbiota. To explore the effect of portions of rice bran that compose soluble fiber vs. those that compose insoluble fiber on the gut microbiota, we used an *in vitro* simulator of the human intestinal microbial ecosystem (SHIME®), mimicking the gut microbiota of three different healthy, adult donors. Genomic and metabolomic analyses were performed to determine differential effects between soluble and insoluble fiber rice bran components as part of a regular diet.

## 2 Materials and methods

### 2.1 Materials

The SHIME® platform was operated following the manufacturer’s guidelines (Prodigest, Belgium) as described previously (25). The defined medium (DM) used in the bioreactors for this experiment was adult MSHIME media with added starch purchased from Prodigest (Ghent, Belgium) (25). Soluble (SF) and Insoluble fractions (ISF) from stabilized Rice Bran were procured from RiceBran Technologies, (Texas, USA). The media used in the control was supplemented with 5 g/L of either SF, ISF, or itself as the control. The DM plus ISF was sonicated for 10 min prior to being autoclaved. Pancreatic Juice (PJ) was made from 12.5 g/L NaHCO_3_ (Sigma-Aldrich, Saint Louis, MO), 6 g/L Bile Salts (BD, Franklin Lakes, NJ), and 0.9 g/L pancreatin (Sigma-Aldrich, Saint Louis, MO). Mucin-agar was prepared using 5% type II porcine mucin (Sigma-Aldrich) and 1% bacterial agar in sterile MilliQ water as described previously (25).

Screened, healthy, homogenized human fecal samples were obtained from OpenBiome (Cambridge, Massachusetts, USA) as described previously (25, 26). In brief, all 3 donor samples were harvested from 3 separate Americans with an average Body Mass Index (BMI), between the ages of 21 and 45 years of age, who had been free of antibiotics for at least 1 year and consumed a typical Western diet. After testing negative for pathogens, fecal samples were homogenized in a glycerol buffer solution to a 10% final concentration and stored at −80°C. Donors used for this study were selected at random from a group of consumers meeting these criteria.

### 2.2 SHIME^®^ *in vitro* experiment

**Figure 1:**
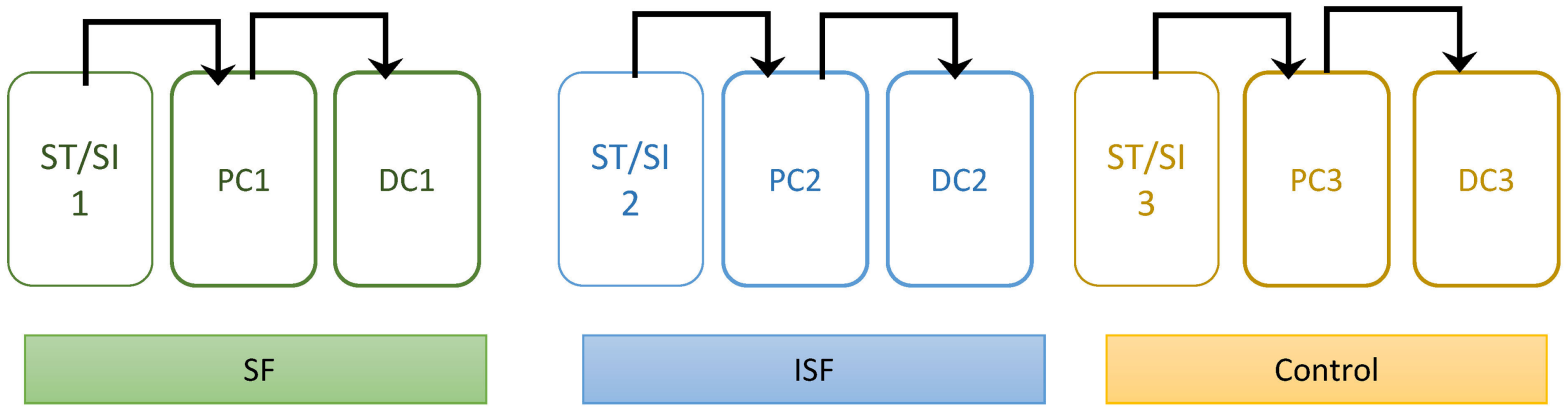
Diagram of Experimental set-up. Each colon has a stomach/small intestine reactor (ST/SI), a proximal colon (PC), and a distal colon (DC). Each colon was also given different treatments: soluble fraction of rice bran (SF), insoluble fraction of rice bran (ISF), and the control. Contents move sequentially through the colon as shown by the arrows.

The SHIME® (Prodigest, Ghent, Belgium) was set up and run similarly to what was described previously (27) with slight modifications. As illustrated in Figure 1, the bioreactors were split into sets of 3, for a total of 3 digestive systems per experimental run. For each set of digestive systems, there were bioreactors simulating the stomach/small intestine, the proximal colon (PC) and the distal colon (DC). The DM and PJ were pumped separately into the stomach/small intestine reactors, and then subsequently into the PC, the DC, and then to waste. Regions are differentiated by volume and pH control, where the stomach (140 mL) was held at pH of 1.9–2.1, the small intestine (200 mL) at a pH of 6.5–7.1, the PC (500 mL) at a pH of 5.6–5.9, and the DC (800 mL) at a pH of 6.6–6.9. Each PC and DC has a luminal (liquid) and mucosal (mucosal agar beads) phase as described previously (25). Inoculum from each donor was tested separately under control, SF, and ISF conditions. Each time, the experiment was performed for 2 weeks to establish community stability, and then 1 additional week for analysis. Luminal and mucosal samples from the cultivars were taken at regular intervals throughout the experiment and stored in a − 80°C freezer prior to analysis.

### 2.3 Next-generation sequencing

DNA was extracted and analyzed at the Microbiome Center, Children’s Hospital of Philadelphia (CHOP) from bacterial pellets obtained at each sampling timepoint using the PowerSoil Pro DNA extraction kit (Qiagen, Hilden, Germany) as described previously (28). DNA was then analyzed using 16S rRNA-tagged gene sequencing. An Illumina Miseq (San Diego, CA, USA) was used to perform the sequencing using a 2 × 250 bp reagent kit following manufacturer guidelines. Libraries were generated as described previously, targeting the V4 region of the 16S rRNA gene (28). The negative controls used were extraction blanks and DNA-free water with the positive control being known amounts of 16S rRNA gene fragments.

### 2.4 Gas-chromatography – mass spectrometry analysis of SCFAs

Analysis and sample preparation was performed as described previously (29). In brief, samples were collected and stored in −80°C, then thawed immediately prior to analysis. After sample preparation, SCFAs were analyzed using GC/MS Shimadzu QP2010 Ultra (Shizmadzu, Columbia, MD, USA) equipped with a Stabilwax-DA column, 30 m, 0.25 mm ID, 0.25 μm (Restek Corporation, Bellefonte, PA, USA). 1 μL of each sample was injected in a 1:20 split mode at 260°C, with an interface temperature of 280°C and an ion source temperature of 220°C. A standard stock solution with a concentration of 5 mg/mL of each of the following: acetic acid, butanoic acid, 2-methylbutanoic acid, valeric acid, propionic acid, isobutanoic acid, hexanoic acid, heptanoic acid, 2-methylvaleric acid, 3-methylvaleric acid, 4-methylvaleric acid, 2-methylvutyric acid, and isovaleric acid was used as described previously (29). The data were acquired with a mass range of *m/z* 25–375 in full scan mode.

### 2.5 Bioinformatics and statistical analysis

Sequence data were processed using QIIME2 (30). Read pairs were processed to identify amplicon sequence variants with DADA2 (31). Taxonomic assignments were generated by comparison to the Greengenes reference database (32), using the naive Bayes classifier implemented in scikit-bio (33). A phylogenetic tree was inferred from the sequence data using MAFFT (34). Similarity between samples was assessed by weighted and unweighted UniFrac distance (35, 36), as well as percent shared species (Jaccard index) and Bray-Curtis distance. PICRUSt2 was used to perform functional estimation based on the 16S rRNA community profiles (37).

Data files from QIIME were analyzed in the R environment for statistical computing, using the QIIMER library, which we developed.^1^ Global differences in bacterial community composition were visualized using Principal Coordinates Analysis. Community-level differences between sample groups were assessed using the PERMANOVA test, which allows sample-sample distances to be applied to an ANOVA-like framework (38). Statistical analysis and visualizations were done using R (v.4.1.3) (39) and the packages tidyverse (40) and ggplot2 (41). Differential estimated enzyme abundance (EC) was calculated using MaAsLin2 (42).

## 3 Results

### 3.1 Both supplement types alter gut microbiota composition

The ability of both soluble (SF) and insoluble (ISF) rice bran supplementation to elicit changes to the gut microbiota was tested using three donors via the SHIME platform. This platform was specifically selected because it allowed us to examine changes to both the proximal (PC) and distal (DC) colons while simultaneously mimicking both the luminal and mucosal communities for each donor. Following stabilization of the SHIME communities (2 weeks) (25), samples were taken for genomic analysis using 16S rRNA tagged sequencing to evaluate changes in community structure. Due to high interindividual variation of the gut microbiota, analyses are shown separated by donor, the region of the gut, and the type of community.

**Figure 2:**
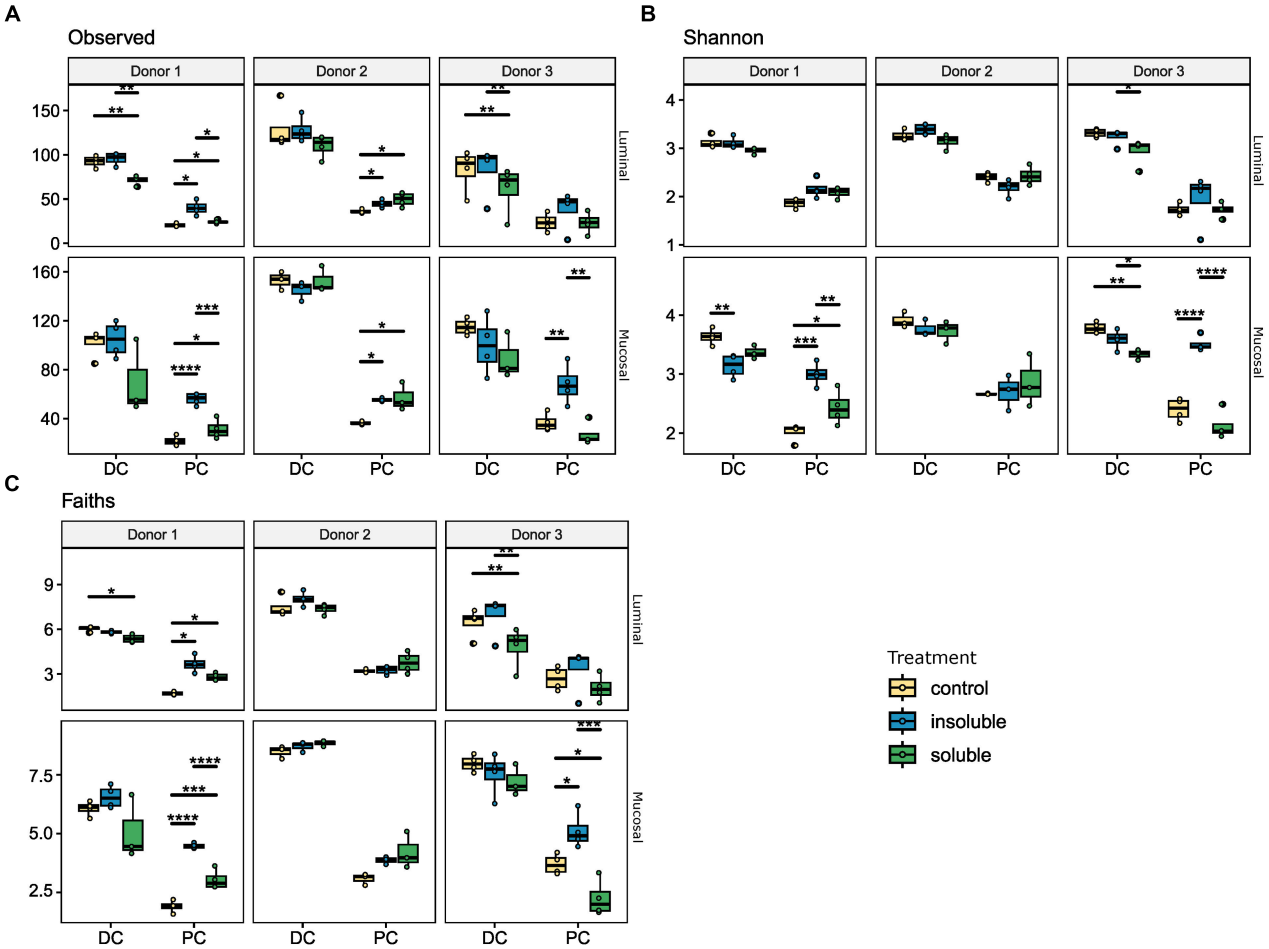
Alpha diversity analysis reveals significant changes with ISF and SF treatment. Donors 1–3, and luminal/mucosal phases are separated for this analysis. For all measures, PC and DC differ significantly. **(A)** Number of observed ASVs, for luminal and mucosal samples, ISF and SF differ significantly; **(B)** Shannon’s diversity index, for mucosal samples SF and ISF differ significantly; **(C)** Faith’s phylogenetic diversity, the luminal phase ISF differs significantly from control and SF, for mucosal phase ISF is significantly different from control. Significance was determined using Tukey’s multiple comparison of means. * = *q* < 0.05; ** = *q* < 0.01; *** = *q* < 0.001; **** = *q* < 0.0001.

First, we considered richness, or the number of unique members of the community. By this measure, as seen in Figure 2A, both the luminal and mucosal communities showed broadly similar patterns of response within each donor, but these patterns varied across donors. Using this measure, we found a significant difference between the SF and ISF treatments, which was particularly strong in the proximal region of the colon with donors 1 and 3. Donor #2, however, did not show a significant difference between any of the conditions. Shannon’s diversity index (Figure 2B) was used to explore the heterogeneity of the microbial community, and we found again that the communities from donor #2 displayed a different pattern than donors 1 and 3. In donors 1 and 3, we found that supplementation with ISF increased diversity of the PC microbial communities to levels higher than that of either SF or the control. However, in the DC luminal communities, ISF supplementation did not affect microbial diversity whereas supplementation with SF caused a decrease in diversity. In the mucosal phase, however, we observed a decrease in diversity with ISF and SF supplementation compared with the control. The final measure of alpha diversity used to analyze these microbial communities was Faith’s phylogenetic diversity (Figure 2C), used to look for changes according to phylogeny and not by taxon alone. Using this measure we found a similar pattern, that donor 2 showed no significant changes in diversity associated with supplementation, whereas donors 1 and 3 showed an increase in diversity with ISF, especially in the PC. Similarly to the other alpha diversity measures discussed, in the DC, we observed a decrease in diversity with SF supplementation compared with control.

**Figure 3:**
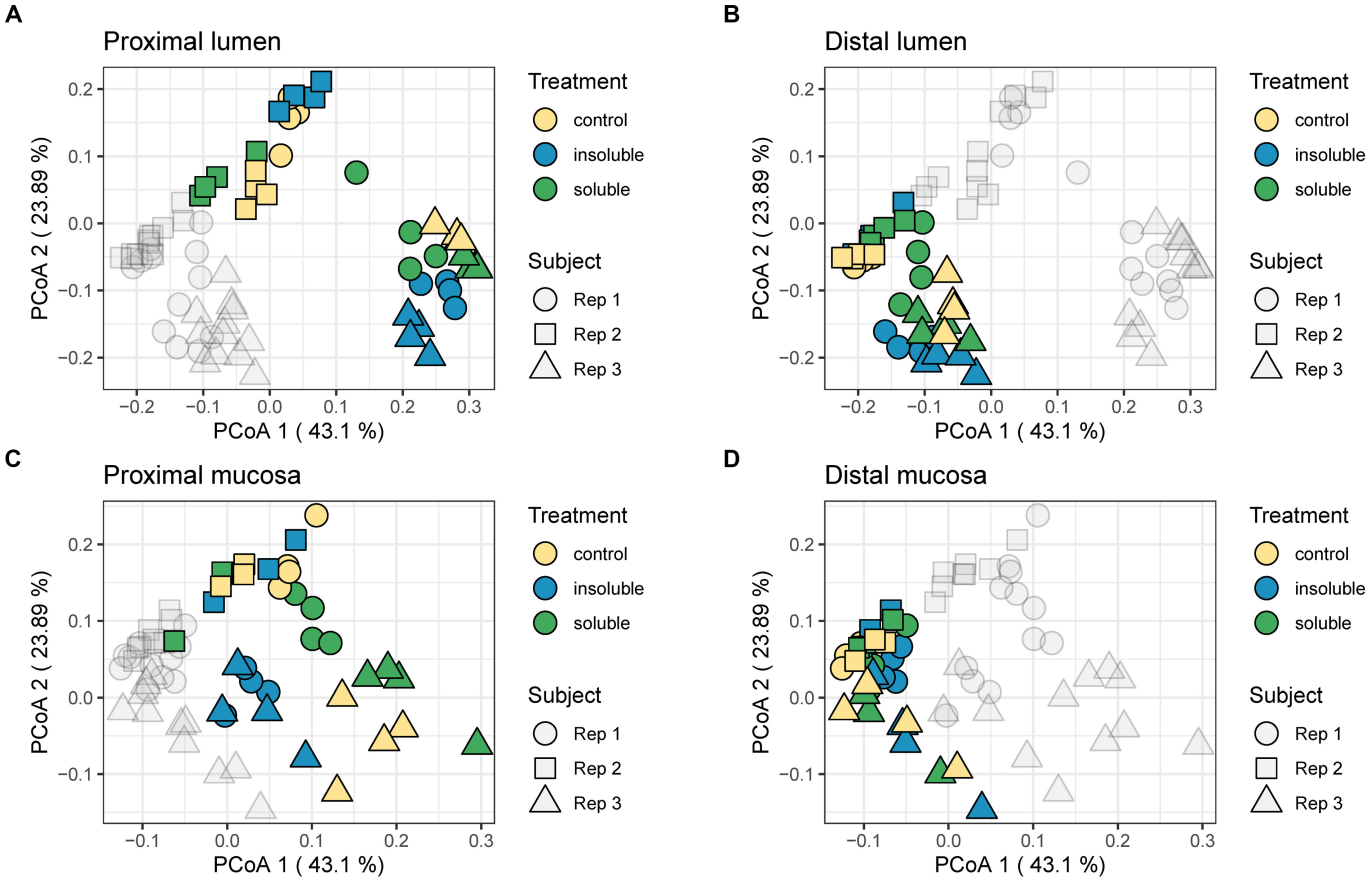
Weighted UniFrac distance shows shifts in the community. **(A)** Proximal lumen; **(B)** Distal lumen; **(C)** Proximal mucosa; **(D)** Distal mucosa. PERMANOVA analysis was used to determine significance. Control treatment differed significantly from ISF (*F* = 1.0078; *q* < 0.05) and SF (*F* = 0.6998; *q* < 0.05); ISF and SF did not differ significantly from each other regardless of region.

Next, beta diversity was evaluated using the weighted UniFrac distance metric and portrayed via principal coordinate analysis (PCoA). In Figure 3, these results were separated by colon region (PC vs. DC) and phase (luminal vs. mucosal). Similarly, to the alpha diversity data, we found that in most cases, donor 2 communities were the most distinct from the other two donors and treatment, indicating that donor is the most important factor in response to supplementation of fiber. In the distal lumen and distal mucosa plots, communities were highly similar, with donor 3 showing the most divergence in the distal mucosal community. The PC communities were more diverse compared with the DC communities with the most changes between treatments found in the proximal mucosal communities. Statistical analysis performed using PERMANOVA found that supplementation with both SF and ISF caused significant changes (*q* < 0.05) in beta diversity compared with control but were not significantly different from each other. Overall, this analysis shows that the largest difference in microbial diversity for these communities is dependent on the donor rather than the type of supplement.

### 3.2 The addition of SF and ISF elicited donor-specific changes to the taxonomic structure of the gut microbiota

**Figure 4:**
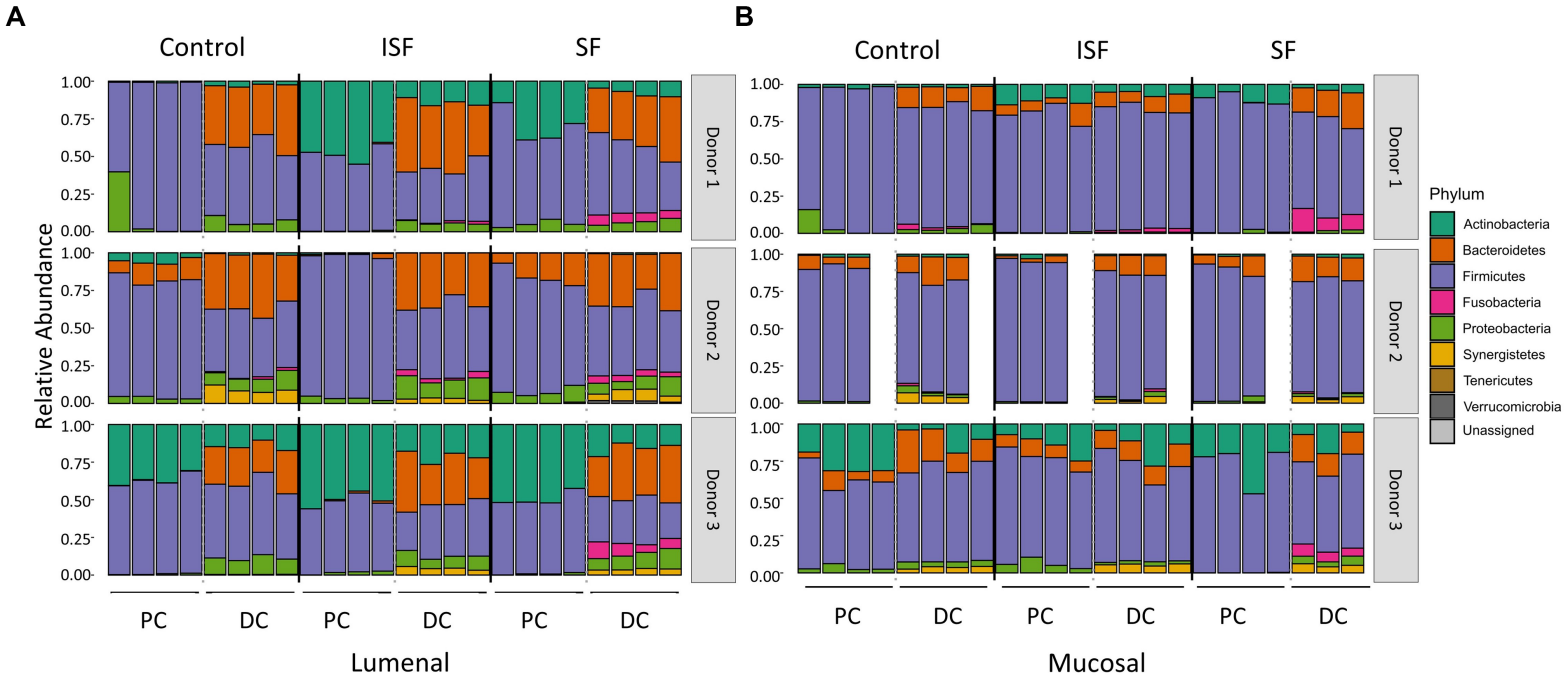
Phyla relative abundance of the luminal and mucosal communities. **(A)** Luminal community; **(B)** mucosal community. Statistical significance (*q* < 0.05) was determined via ANOVA.

Although the results of UniFrac distances demonstrated that both SF and ISF elicited shifts in the microbial communities to the same extent, it did not provide information on which taxa were responding to these supplements. Using sequencing data from above, changes to the addition of SF and ISF were determined based on the relative abundance of the microbiota at the phylum level. Similar to the results of alpha diversity and UniFrac distances, supplementation with SF and ISF caused donor-dependent changes to the taxonomic structure at the phylum level (Figure 4). At this level, we found that both treatments caused a notable but not statistically significant increase in the *Actinobacteria* in the donor 1 communities, as well as a significant (*q* < 0.0001) increase in *Fusobacteria* in the DC in the lumen. This increase in *Fusobacteria* was also apparent in donor 3, but only with SF treatment (*q* < 0.05). In the mucosal phylum level analysis (Figure 4B), there is a similar pattern, though this part of the gut microbial community is more highly dominated by the *Firmicutes* than the luminal counterpart.

**Figure 5:**
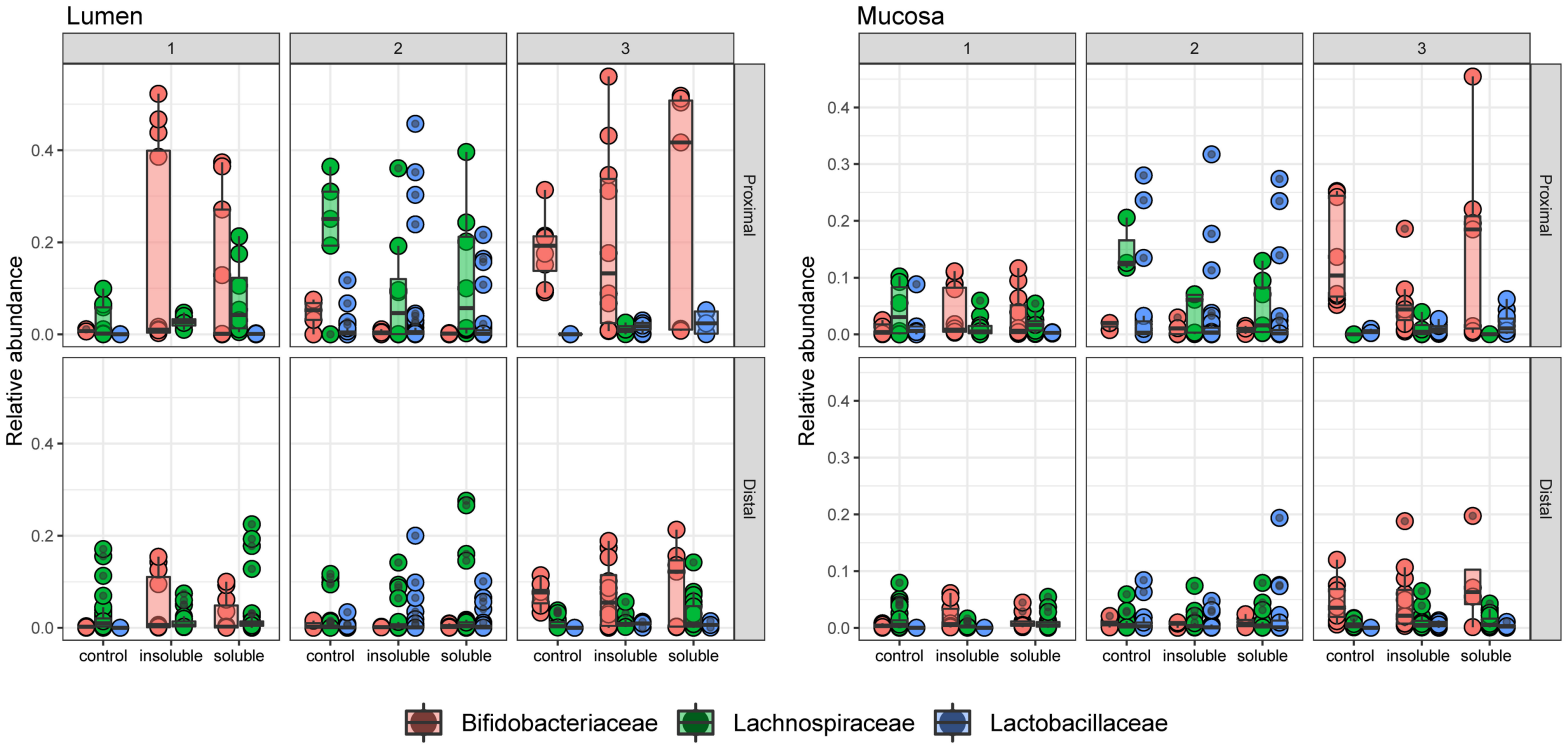
Relative abundance of 3 key taxa at the family level. Relative abundance of Bifidobacteriaceae, Lachnospiraceae, and Lactobacillaceae in each community. Significance determined via Tukey’s multiple comparison of means and shown in Supplementary Table S1.

Next, we examined the abundances of three bacterial families (*Bifidobacteriaceae*, *Lachnospiraceae*, and *Lactobacillaceae*) that are known to contribute to SCFA production and fermentation of fibers and are likely to have overlapping roles in metabolism (Figure 5, statistical significance shown in Supplementary Table S1). We observed that *Bifidobacteriaceae* is a key member of the community in donors 1 & 3 and is significantly increased with ISF and SF compared with control in the lumen for donor 1 (*q* < 0.0005, *q* < 0.05, respectively). In the mucosa, *Bifidobacteriaceae* increased significantly with SF and ISF treatment, *Lachnospiraceae* decreased significantly with ISF and SF treatment (*q* < 0.0005; *q* < 0.05), and *Lactobacillaceae* (*q* < 0.0005) decreased significantly with SF treatment in the PC of donor 1. Donor 2 exhibited a significant decrease in *Bifidobacterium* with for ISF (*q* < 0.005) and SF (*q* < 0.0005) treatment in both the luminal and mucosal community PCs, though the overall *Bifidobacterium* content in the gut microbiome is low in donor 2. Donor 2 had a larger abundance of both *Lachospiraceae* and *Lactobacillaceae* than the other two donors but did not experience a significant change in abundance of either of these families with either SF or ISF treatment. Donor 3 did not exhibit significant changes in *Bifidobacterium* abundance based on supplement type, however, donor 3 did have a substantial abundance of *Bifidobacteriaceae* in both the lumen and the mucosa, similar to donor 1. *Lactobacillaceae* and *Lachnospiraceae* did increase significantly (*q* < 0.0005) with ISF treatment in the luminal and mucosal phases of donor 3’s PC, but it is also worth noting that these families have low abundance in donor 3 overall.

**Figure 6:**
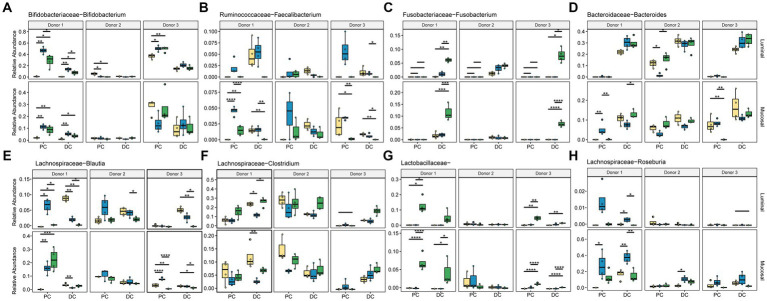
Relative abundance of significant taxa. Colors are as found in previous figures: yellow indicates control, blue indicates ISF, green indicates SF. Significance was determined using ANOVA and is shown in Supplementary Table S2. **(A)** *Bifidobacteriaceae Bifidobacterium*; **(B)** *Ruminococcaceae Faecalibacterium*; **(C)** *Fusobacteriaceae Fusobacterium*; **(D)** *Bacteroidaceae Bacteroides*; **(E)** *Lachnospiraceae Blautia*; **(F)** *Lachnospiraceae Clostridium*; **(G)** *Lactobacillaceae*; **(H)** *Lachnospiraceae Roseburia*. * = *q* < 0.05; ** = *q* < 0.01; *** = *q* < 0.001; **** = *q* < 0.0001.

This data was also used to look more closely at specific taxa known to ferment dietary fibers or that showed significant changes in response to these different supplementation options (Figure 6; Supplementary Table S2). As expected, changes seen at this level are donor-, region-, and phase-dependent. *Bifidobacterium* (Figure 6A) significantly increased in relative abundance with ISF in the PC regions for donor in the luminal phase (*q* < 0.0005) and the mucosal phase (*q* < 0.0005), with a smaller, non-significant increase in the DC as well. In the mucosal phase, ISF decreased in relative abundance of *Bifidobacterium* in comparison with control for donor 3, however this change was not statistically significant. Treatment with SF also increased *Bifidobacterium* in the luminal phase of the PC in donor 1. Conversely, ISF (*q* < 0.005) and SF (*q* < 0.0005) both caused a significant decrease in *Bifidobacterium* abundance in the PC of donor 2, however the starting abundance of *Bifidobacterium* was already low.

*Faecalibacterium* (Figure 6B) relative abundance significantly increased with ISF treatment only in donors 1 and 3 which was evident in the luminal PC. SF, on the other hand, significantly increased the abundance of *Fusobacterium* in the DC of donor 1 in both the luminal and mucosal phases (Figure 6C). The relative abundance of *Bacteroides* (Figure 6D), one of the more abundant members of the gut microbiota, did not change significantly with either fiber treatment.

The *Lachnospiraceae* family had the most members that changed significantly with fiber supplementation, including *Blautia*, and *Clostridium*. *Blautia* (Figure 6E) was overall more abundant in the PC but increased in abundance with ISF treatment in the luminal phase (*q* < 0.005) and mucosal phase (*q* < 0.05) for donor 1. With SF treatment, *Blautia* saw an increase with the PC of donor 1 (*q* < 0.05) and the DC of donor 3 (*q* < 0.05). *Clostridium* (Figure 6F) abundance significantly increased with ISF treatment for donor 1 in the DC, luminal and mucosal phases (*q* < 0.05), and it increased abundance with SF treatment in the DC of donor 3, luminal and mucosal phases (*q* < 0.0005). *Roseburia*, on the other hand, appears to reside only in the mucosal phase of the colons, and did not change in a statistically significant way. For this taxon (Figure 6H), we found that treatment with ISF increased abundance over control and SF in both the PC and the DC with donor 1, but in the other two donors was mostly present in the DC. Donor 1 also showed an increased in abundance of *Roseburia*, but only in the PC, and again this change was not significant. Another category, the otherwise un-categorized *Lactobacillaceae* genera (Figure 6G), showed significant increases in abundance with SF treatment, however, this is largely the case with donor 1 and donor 3 (*q* < 0.0005) in the PC. However, for donor 1 there was a smaller, but still significant increase in the DC as well (*q* < 0.05). Overall, our data demonstrates that responses to these rice bran supplements are highly dependent on individual taxa and donor communities.

### 3.3 Short-chain fatty analysis reveals interindividual changes

**Figure 7:**
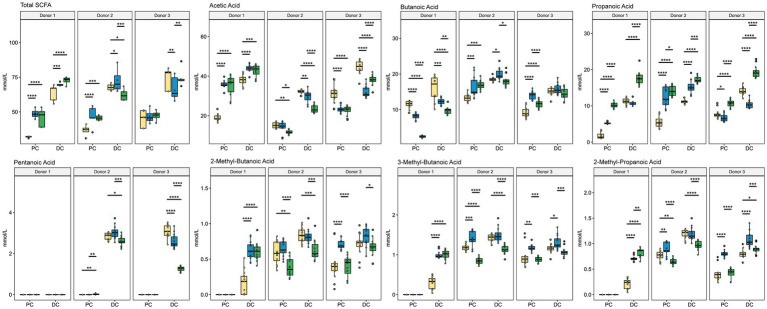
SCFA Concentration. Colors are as found in previous figures: yellow indicates control, blue indicates ISF, and green indicates SF. Significance was determined using Tukey’s multiple comparisons of means (*q* < 0.05) shown in Supplementary Table S3. * = *q* < 0.05; ** = *q* < 0.01; *** = *q* < 0.001; **** = *q* < 0.0001.

To understand how the gut microbiota changed functionally due to supplementation, we performed GC–MS/MS to analyze the concentration of SCFAs for each donor under each condition (Figure 7; Supplementary Table S3). As expected, overall concentrations of SCFAs increased in the DC compared with the PC regardless of treatment type. Interestingly, donors 2 and 3 had similar responses to treatment with respect to SCFA concentrations, whereas the response of donor 1 was much more unique. For example, acetic acid increased with both ISF and SF supplementation for donor 1 (*q* < 0.0005) but decreased for donors 2 and 3 (*q* < 0.0005). Propanoic acid decreased in response to ISF in the PC of donor 1 (*q* < 0.0005) and the DC (*q* < 0.05) of donor 3 but increased for donor 2 in both the PC and DC (*q* < 0.0005). Both treatment groups increased butanoic acid for donors 2 and 3 in the PC, though only SF with donor 3 showed a significant change (*q* < 0.0005) but SF treatment decreased butanoic acid in concentration for donor 1 (*q* < 0005). The concentration of pentanoic acid was very low in donor 1 but was present in the DC of donors 2 and 3. Supplementation with SF decreased pentanoic acid in both donors, but only significantly so with donor 3 (*q* < 0.05). ISF caused an increase in pentanoic acid concentration in donor 2, (though this change was not significant) while decreasing the concentration in donor 3 (*q* < 0.05). Branched-chain short-chain fatty acids (BCSCFAs) 3-methylbutanoic acid, 2-methylpropanoic acid, and 2-methylbutanoic acid had similar responses to the treatments. In donor 1, these compounds were only present in the DC, and both rice bran supplements increased the concentration of all three BCSCFAs (*q* < 0.0005). In donor 2, SF decreased the concentrations of the 3 BCSCFAs in both the PC and the DC, whereas 3-methylbutanoic acid and 2-methylpropanoic acid increased in the PC with ISF supplementation, however these changes were not statistically significant. With donor 3, all 3 BCSCFAs increased in response to ISF in both the PC and the DC (significance shown in Supplementary Table S3), whereas the SF treatment remained the same as control in the PC but slightly decreased in the DC. This data, along with that of the microbial community diversity and taxon abundance confirms that the response of the gut microbiota to SF and ISF supplementation depends largely on the native microbial community.

### 3.4 Enzymes for starch metabolism may come from different players depending on supplement type

**Figure 8:**
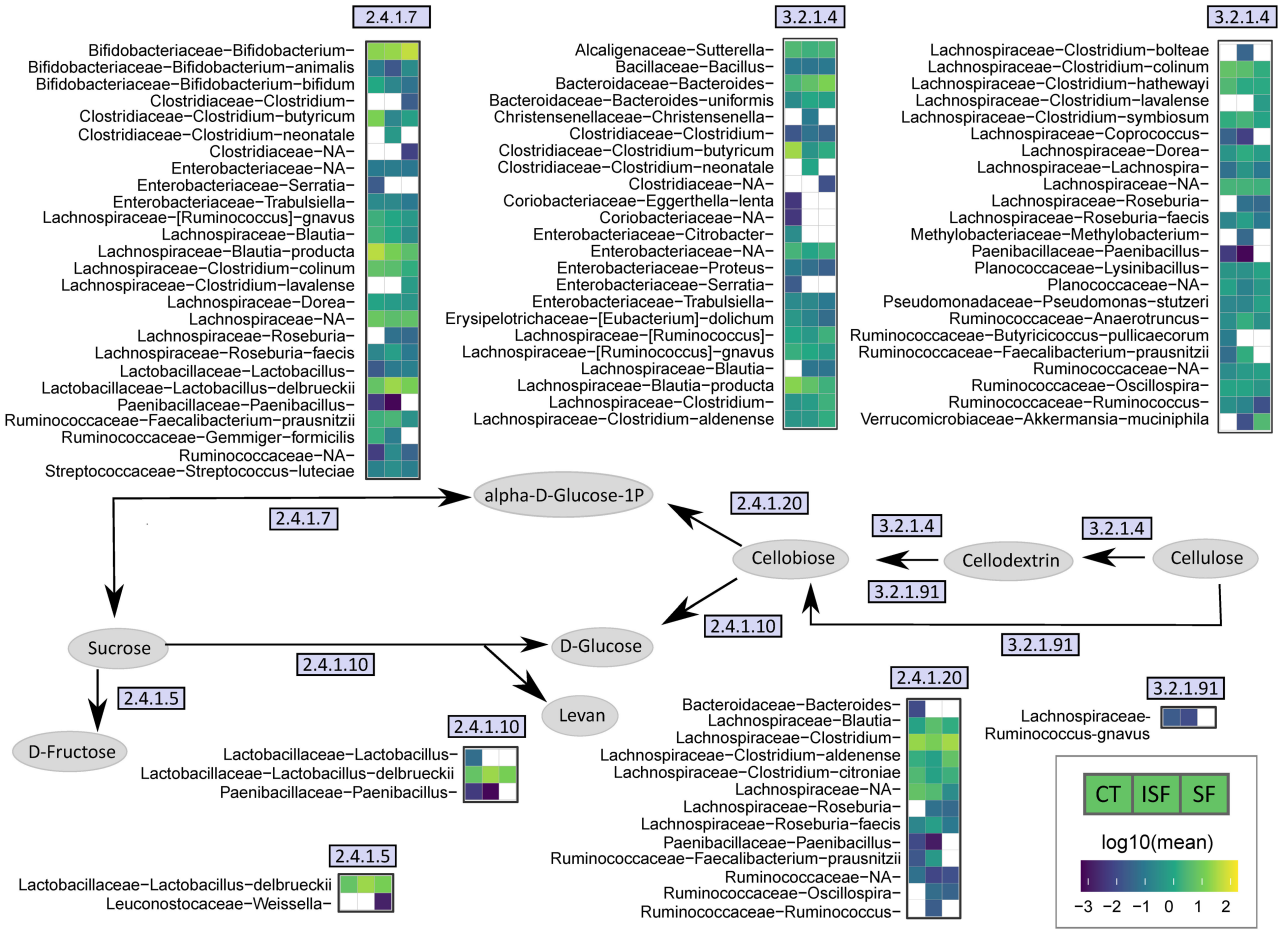
Simplified cellulose metabolism pathway. This figure illustrates a part of the cellulose metabolism pathway by the gut microbiota, using EC numbers to identify genes associated with enzymes involved in the process that are significantly changed with either ISF or SF supplementation. Heatmaps demonstrate specific taxa that are associated with each enzyme and may contribute to its activity.

Analysis of community structure and SCFAs portrayed high interindividual variability in the response of the gut microbiota to both SF and ISF. As each microbiome is distinct, this variability was somewhat expected, however, we hypothesized that while the taxa and functional response may have been dependent on the donors’ starting community structure, genetic pathways enriched due to SF and ISF would be similar and not driven by interindividual variability. Therefore, PICRUSt2 was used to predict the presence of genes that may be responsible for cellulose metabolism within these gut microbial communities. A portion of this metabolic pathway is shown in Figure 8, illustrating enzymes whose abundance was significantly altered by either SF or ISF treatment. The taxa relevant to each enzyme are shown in adjacent heatmaps. In this figure, *Lachnospiraceae* is a major contributor to the enzymes involved in the conversion of cellulose to cellobiose, and for some of the *Lachnospiraceae* family members, they are only associated with specific treatment types. For example, *Coprococcus* is only present with control and ISF use and *Roseburia* is present with both ISF and SF use, but not present for the control for cellulase (EC3.2.1.4).

*Lactobacillus delbrueckii* is associated with sucrose phosphorylase (EC 2.4.1.7), levansucrase (EC 2.4.1.10), and dextransucrase (EC 2.4.1.5), and has a greater abundance with ISF and SF compared with control. *Bifidobacterium* appears to be more represented with SF than with control or ISF with sucrose phosphorylase (EC 2.4.1.7). From our relative abundance data shown above, we know that *Bifidobacterium* has a greater presence with donors 1 and 3, while *Lactobacillus delbrueckii* has a greater abundance with donor 2 (Figure 5). This indicates that *Bifidobacterium* and *Lactobacillus delbrueckii* may have overlapping roles in starch metabolism, and that this functional redundancy is likely what allows for gut microbiota from different donors to ferment the same dietary fibers.

## 4 Discussion

Continued industrialization and westernization of communities around the world has led to an increase in gut health disorders, including Crohn’s disease and other irritable bowel diseases, as well as related problems such as obesity, diabetes, and autoimmune disorders (43, 44). Many of these changes are thought to be due to the increase in consumption of processed foods with a reduction of whole foods in the diet, especially high-fiber, nutrient-dense vegetables, and whole grains. These dietary changes that have occurred over time have gradually lessened the diversity of the average person’s gut microbial community, a change that continues with each subsequent generation (45). In this study, we used by-products of rice processing in the form of rice bran fractionated into soluble and insoluble parts, largely fibers, to find how these supplements can shape the gut microbiota over time. We used the SHIME® platform to determine how the same starting inoculum from 3 donors responded to growth media supplemented with either fraction of the rice bran fiber compared with the control.

Through genomic analysis, this study found that supplementation of both SF and ISF caused changes in the gut microbial community but that the nature and direction of these changes varied by donor. Surprisingly, we observed a greater increase in alpha diversity with ISF than with SF compared with control. While the common understanding is that soluble fiber is easily fermented by the gut microbiota while insoluble fiber is not, our findings that the ISF fractions change the gut microbiota are not unheard of considering other recent research looking into the effects of insoluble fiber from other staple crops on the gut microbiota (14, 16). In previous work, researchers found that insoluble fiber from soybeans increased the diversity of the mouse gut microbiota, the relative abundance of specific taxa, and the production of SCFAs, indicating that fermentation of these insoluble fibers was indeed occurring (14). In an *in vitro* study using human feces as the starting material, other researchers found that the use of insoluble fibers from soy husks modified the gut microbiota via an increase in alpha diversity and an increase in specific taxa, with a notable increase in *Bifidobacterium* (16). In this study, we observed a similar increase in alpha diversity with ISF, however, changes in *Bifidobacterium* abundance were more variable and were highly dependent on donor, community type, and colon region (PC or DC). *Bifidobacterium* are an integral part of a healthy gut microbiota, and members of the genus are frequently used as probiotics and as part of functional foods to improve gut health (46). Here, we found that for Donors 1 and 3, *Bifidobacterium* was either unchanged or increased in both the PC and DC regions of the lumen, however, *Bifidobacterium* abundances were higher in the PC than the DC, suggesting that their preferred substrates are available in the PC and have likely been depleted before reaching the DC. This pattern holds in the mucosal for donors 1 and 3 except for the PC of donor 3, where *Bifidobacterium* abundances were lower with treatment compared to controls, however, it is important to note that as these are relative abundances, these decreases could reflect an increase in other taxa, rather than a decrease in Bifidobacteria. Interestingly, Bifidobacteriaceae was almost completely absent in donor 2 and appeared to be replaced by the members of the Lachnospiraceae. For donor 2, which did not have a large community presence of *Bifidobacterium*, we did find that other family members also involved in fiber metabolism are likely to be involved, most notably *Lactobacillus delbrueckii,* which has been shown previously to be involved in inulin metabolism via the ability to uptake inulin and degrade it as a source of carbon for growth (47). *L. delbrueckii* is a probiotic strain frequently used in culturing yogurt, which may suggest different dietary habits of the donors used in this study (48).

Another taxon that increased significantly with ISF treatment in our study was *Faecalibacterium*, which increased significantly in abundance in the PC for all donors. Species within *Faecalibacterium* are associated with a lower incidence of IBD and other diseases related to colonic inflammation and have been demonstrated to produce SCFAs, including acetate and butyrate (49). The abundance of *Faecalibacterium* has been significantly increased in previous studies with the use of other dietary fibers, like inulin in human studies (50). The increase of this genus with ISF treatment may contribute to the increase in acetic acid and butanoic acid seen in some of our donors as well. These findings, that similar members of the gut microbiota are involved in degradation of additional fiber types, like those found in rice bran, highlight that there is functional redundancy integral to the gut microbiota. This is likely what allows for beneficial effects from an increase in dietary fiber for most individuals, despite individual differences in the gut microbial community.

The family with the most significant changes in abundance with either SF or ISF supplementation was the *Lachnospiraceae* family. This family is a major contributor to the production of SCFAs, greater production of which is associated with better health outcomes (51–53). However, the impact of various players within *Lachnospiraceae* on human health is more controversial. *Blautia* and *Roseburia*, two genera that increased with ISF and SF compared with control, are major SCFA producers and have been previously demonstrated as beneficial for their anti-inflammatory properties and their contribution to the health of the immune system (54, 55).

The fermentation of dietary fibers by the gut microbiota produces SCFAs as microbial metabolites that are important for gut and overall health. For example, acetic acid has been shown to alleviate aging symptoms in gut health of mice (56). The production of butanoic acid in particular is known to benefit tight-junction formation in the gut, and therefore increase barrier integrity of the gut (57). Butanoic acid is also linked to the production of mucus by the goblet cells of the large intestine (58). Propanoic acid has been shown to have anti-inflammatory effects, to improve insulin sensitivity, and improve satiety (59). Our study demonstrated increases in SCFA production, suggesting broader trends, however many of these changes were subject to interindividual variability. For example, the reactors from all donors had significantly increased propionate concentrations in the SF relative to controls in both the PC and DC, while the propionate response to ISF was mixed. Acetic acid production displayed a more pronounced donor effect, with significant increases in acetic acid production in ISF and SF relative to controls in donor 1 reactors, with the opposite effect in donor 3 reactions. Interestingly in the case of donor 3 reactors, the acetic acid production in the control samples was already among the highest measured concentrations. Similarly, for butanoic acid, donors 2 and 3 had increases or stable concentrations of butanoic acid in the supplemented samples relative to controls with an opposite trend for donor 1 where the control samples had the highest concentrations. As with acetic acid, in those communities where the concentrations were highest in the control samples, it’s possible that those communities were already predisposed to certain pathways. The extent of inter-individual variability in gut microbiota is well known and likely underlies the complex SCFA results shown here. This variability can be seen in taxa known to be associated with SCFA production and mirrors the changes in the population of SCFA producing members of the gut microbiota. Combined with our results, we interpret the increase in these families to indicate that supplementation with both SF and ISF may have beneficial effects on the gut microbiota.

Overall, this study determined that both soluble and insoluble fractions of fiber from rice bran elicited a microbial response, however, the nature of the response varied among the donor and colon regions. This is likely due to the different initial states of the donors’ microbial communities and agrees with the well-known phenomenon of a high level of variability among individuals and the gut microbiome (60). These nuanced variations are important to consider and underpin the growing fields of personalized nutrition and personalized medicine and underscores the need for fine-grained sampling across donors and colon regions and additional such studies with larger cohorts, however, overall, these results suggest a potential use of fiber from rice bran as a functional food which merits continued exploration.

## Data availability statement

The metabolite analysis data is available as supplementary material, found in Supplemental Table 4. The original sequencing contributions presented in the study are publicly available. This data can be found at: https://www.ncbi.nlm.nih.gov/bioproject/PRJNA1032541/.

## Ethics statement

Ethical approval was not required for the studies involving humans because fecal samples were purchased from Openbiome, and acquired from a by-product of routine care or industry. The studies were conducted in accordance with the local legislation and institutional requirements. Written informed consent to participate in this study was not required from the participants or the participants’ legal guardians/next of kin in accordance with the national legislation and the institutional requirements.

## Author contributions

KM: Conceptualization, Data curation, Formal analysis, Investigation, Methodology, Writing – original draft, Writing – review & editing. LL: Conceptualization, Investigation, Writing – review & editing. JB: Conceptualization, Investigation, Methodology, Writing – review & editing. AN: Conceptualization, Data curation, Formal analysis, Methodology, Validation, Visualization, Writing – original draft, Writing – review & editing. JF: Investigation, Methodology, Writing – review & editing. KB: Data curation, Formal analysis, Writing – review & editing. WH: Data curation, Formal analysis, Writing – review & editing. SJ: Investigation, Methodology, Writing – review & editing. AM: Investigation, Methodology, Writing – review & editing.

## Footnotes

^1^http://cran.r-project.org/web/packages/qiimer

## Supplementary material

Supplementary Table 4 Gas-Chromatography-Mass Spectrometry analysis of short-chain fatty acid content raw data in mmol/L. RB1/2/3 indicates donors 1, 2, 3; UC indicates proximal colon, DC indicates distal colon.
