# Supplementary material for: Supplementation with soluble or insoluble rice-bran fibers increases short-chain fatty acid producing bacteria in the gut microbiota in vitro
Source: Front Nutr. 2024 May 10;11:1304045. doi: 10.3389/fnut.2024.1304045 (PMC11116651; doi:10.3389/fnut.2024.1304045)
Supplement: Supplementary Table 4 — Gas-Chromatography-Mass Spectrometry analysis of short-chain fatty acid content raw data in mmol/L. RB1/2/3 indicates donors 1, 2, 3; UC indicates proximal colon, DC indicates distal colon. [file Table_1.DOCX]

Supplemental Table 1: correlated to Figure 5, significance was determined using Tukey’s multiple comparison of means with a 95% confidence interval and an adjusted p value. * q< 0.05, ** q < 0.005, *** q< 0.0005.

Supplemental Table 2: Correlated to Figure 6, significance was determined using Tukey’s multiple comparison of means with a 95% confidence interval and an adjusted p value. * q< 0.05, ** q < 0.005, *** q< 0.0005.

|  |  | lumenal | | | | | | mucosal | | | | | |
| --- | --- | --- | --- | --- | --- | --- | --- | --- | --- | --- | --- | --- | --- |
|  |  | Donor 1 | | Donor 2 | | Donor 3 | | Donor 1 | | Donor 2 | | Donor 3 | |
|  |  | PC | DC | PC | DC | PC | DC | PC | DC | PC | DC | PC | DC |
| Bifidobacteriaceae Bifidobacterium | ISF vs control | *** |  | ** |  |  |  | *** |  | ** |  |  |  |
|  | SF vs control | * |  | *** |  |  |  | * |  | *** |  |  |  |
|  | ISF vs SF |  |  |  |  |  |  |  |  |  |  |  |  |
| Ruminococcaceae Faecalibacterium | ISF vs control | ** |  |  |  | *** |  | ** |  |  |  | *** |  |
|  | SF vs control |  | * |  |  |  |  |  | * |  |  |  |  |
|  | ISF vs SF |  | ** |  |  |  |  |  | ** |  |  | *** |  |
| Fusobacteriaceae Fusobacterium | ISF vs control |  |  |  |  |  |  |  |  |  |  |  |  |
|  | SF vs control |  | *** |  |  |  | *** |  | *** |  |  |  | *** |
|  | ISF vs SF |  | *** |  |  |  | *** |  | *** |  |  |  | *** |
| Bacteroidaceae Bacteroides | ISF vs control |  |  |  |  |  |  |  |  |  |  |  |  |
|  | SF vs control |  |  |  |  |  |  |  |  |  |  |  |  |
|  | ISF vs SF |  |  |  |  |  |  |  |  |  |  |  |  |
| Lachnospiraceae Blautia | ISF vs control | ** |  |  |  |  |  | * |  |  |  |  |  |
|  | SF vs control | * |  |  |  |  | * | * |  |  |  |  | * |
|  | ISF vs SF |  |  |  |  | * |  |  |  |  |  | * |  |
| Lachnospiraceae Clostridium | ISF vs control |  | * |  |  |  |  |  | * |  |  |  |  |
|  | SF vs control |  |  |  |  |  | *** |  |  |  |  |  | *** |
|  | ISF vs SF |  | * |  |  |  | *** |  | * |  |  |  | *** |
| Lactobacillaceae | ISF vs control |  |  |  |  |  |  |  |  |  |  |  |  |
|  | SF vs control | *** | * |  |  | *** |  | *** | * |  |  | *** |  |
|  | ISF vs SF | *** | * |  |  | *** |  | *** | * |  |  | *** |  |
| Lachnospiraceae Roseburia | ISF vs control |  |  |  |  |  |  |  |  |  |  |  |  |
|  | SF vs control |  |  |  |  |  |  |  |  |  |  |  |  |
|  | ISF vs SF |  |  |  |  |  |  |  |  |  |  |  |  |

Supplemental Table 3 : correlated to Figure 7, significance was determined using Tukey’s multiple comparison of means with a 95% confidence interval and an adjusted p value. * q< 0.05, ** q < 0.005, *** q< 0.0005.

|  |  | lumenal | | | | | |
| --- | --- | --- | --- | --- | --- | --- | --- |
|  |  | Donor 1 | | Donor 2 | | Donor 3 | |
|  |  | PC | DC | PC | DC | PC | DC |
| Total SCFA | ISF vs control | *** | *** | *** | *** | *** | *** |
|  | SF vs control | *** | *** | *** | *** | *** | *** |
|  | ISF vs SF |  | *** |  | *** |  | *** |
| Total BCSCFA | ISF vs control | *** | *** | *** | *** | *** | *** |
|  | SF vs control | *** | *** | *** | *** | *** | *** |
|  | ISF vs SF |  |  |  | *** |  | *** |
| Acetic Acid | ISF vs control | *** | * |  |  | ** | *** |
|  | SF vs control | *** |  |  | *** | *** | ** |
|  | ISF vs SF |  |  |  |  |  | ** |
| Propanoic Acid | ISF vs control | *** |  | *** |  |  | * |
|  | SF vs control | *** | *** | *** | * | * | *** |
|  | ISF vs SF | *** | *** |  |  | ** | *** |
| Propanoic Acid, 2-methyl | ISF vs control |  | *** |  |  | *** | * |
|  | SF vs control |  | *** |  | * |  |  |
|  | ISF vs SF |  | * | * | * | *** |  |
| Butanoic Acid | ISF vs control |  | ** |  |  | *** |  |
|  | SF vs control | *** |  |  |  |  |  |
|  | ISF vs SF | ** |  |  |  |  |  |
| Butanoic Acid, 3-methyl | ISF vs control |  | *** |  |  | * |  |
|  | SF vs control |  | *** | * |  |  |  |
|  | ISF vs SF |  |  | *** | ** | ** |  |
| Butanoic acid, 2-methyl | ISF vs control |  | *** |  |  | ** |  |
|  | SF vs control |  | *** |  |  |  |  |
|  | ISF vs SF |  |  | * |  | * |  |
| Pentanoic Acid | ISF vs control |  |  |  |  |  | * |
|  | SF vs control |  |  |  |  |  | *** |
|  | ISF vs SF |  |  |  | * |  | *** |
